# Imaging Canine Post‐Trabecular Aqueous Outflow Pathways: Effect of Acute Intraocular Pressure Elevation in Normal Eyes

**DOI:** 10.1111/vop.70080

**Published:** 2025-09-22

**Authors:** Odalys Torné, Jacob P. Nilles, Andrew L. Smith, Kevin C. Snyder, Kazuya Oikawa, Julie A. Kiland, Mary R. Telle, Mark Banghart, Gillian J. McLellan

**Affiliations:** ^1^ Department of Surgical Sciences, School of Veterinary Medicine University of Wisconsin‐Madison Madison Wisconsin USA; ^2^ Department of Ophthalmology and Visual Sciences, School of Medicine and Public Health University of Wisconsin‐Madison Madison Wisconsin USA

**Keywords:** aqueous angiography, distal aqueous outflow, dog, glaucoma, OCT, scleral venous plexus

## Abstract

**Purpose:**

To investigate the relationship between increased intraocular pressure (IOP) and the structure of the post‐trabecular aqueous outflow tract of dogs.

**Methods:**

Ex vivo aqueous angiography (AA) and anterior segment optical coherence tomography (OCT) were performed concurrently in 19 normal canine eyes, following cannulation and intracameral infusion of 2.5% fluorescein and/or indocyanine green (ICG) after maintaining eyes at physiologic IOP (10–25 mmHg) for 30 min and/or elevated IOP (55–68 mmHg) for 30 or 120 min. Scleral lumen heights (SLH) of vascular profiles in OCT scans were measured by Image J, and values analyzed in linear regression models. In a subset of 10 eyes, each quadrant was subjectively characterized as being either a high‐flow or low‐flow region based on angiographic signal intensity at 2 or 10 min post‐tracer infusion.

**Results:**

Segmental AA outflow signal correlated with lumens seen on OCT scleral line scans in healthy canine eyes at physiologic IOP. These lumens were also observed at elevated IOP but appeared collapsed in profile. After both 30 and 120 min of IOP elevation, SLH was significantly reduced (*p* < 0.0001). Large vessels tended to collapse more than small vessels after IOP elevation.

**Conclusion:**

Acute IOP elevation resulted in a significant reduction in SLH in healthy canine eyes, and the number of AA determined high flow regions was reduced. Future studies to understand the nature and timing of this pressure‐dependent change in the post‐trabecular outflow pathway in glaucomatous eyes, including its potential reversibility, will be crucial in developing novel diagnostic tools and treatments for canine glaucoma.

## 1 Introduction

Goniodysgenesis‐related primary angle closure glaucoma (PACG) is a common cause of blindness in dogs and is a painful condition with a guarded prognosis for retention of vision and the affected globe despite medical and surgical management [1]. Many pure‐breed dogs are known to be at risk for developing PACG, which is considered to have a heritable basis, and the prevalence of the disease in some canine breeds far exceeds that in the general canine population [2]. Homeostasis of intraocular pressure (IOP) is maintained by an intricate balance between production and outflow of aqueous humor [3]. Medical management using osmotic agents, carbonic anhydrase inhibitors, beta‐blockers, and prostaglandin analogues can lower IOP in PACG; however, the extent of IOP control is widely variable [1, 4, 5]. Furthermore, surgical treatments involving aqueous humor shunts and cyclodestructive procedures often fail to adequately regulate IOP long term [6, 7, 8]. Thus, there is a critical need for greater understanding of the underlying complex pathophysiology of IOP dysregulation in dogs with PACG.

In normal canine eyes, the bulk of aqueous humor outflow occurs via the conventional pathway, through the trabecular meshwork (TM) and the vacuolating endothelium of the discontinuous and branching angular aqueous plexus (AAP; analogous to Schlemm's canal of humans) and a series of interconnected collector channels to the distal vessels of the episcleral veins and scleral venous plexus [9, 10, 11, 12]. Aqueous Angiography (AA) is a dynamic aqueous outflow imaging technique that provides real‐time information on these distal, post‐trabecular structures of the conventional outflow pathway and has been used both ex vivo and in vivo under physiologic conditions in human and canine eyes [13, 14, 15, 16].

Our previous ex vivo study identified collapsed scleral vascular channels in canine eyes with PACG [17]. However, whether these results represented a cause or effect of elevated IOP was not established. The goal of this study was to determine the effect of acute, controlled non‐physiologic increases in IOP, consistent with IOPs in spontaneous canine PACG, on the structure of the distal, post‐trabecular aqueous humor outflow tract in normal ex vivo canine eyes imaged by AA and OCT.

## 2 Materials and Methods

### 2.1 Imaging

Aqueous angiography and anterior segment optical coherence tomography (OCT) were performed ex vivo in 19 globes from 10 healthy dogs (6–14 months of age). Enucleated eyes were obtained immediately post‐mortem with adnexa intact from experimental Beagles that had been euthanized for non‐ocular studies unrelated to the current study that were conducted in compliance with approved Institutional Animal Care and Use Committee protocols. Within 4 h post‐mortem, ex vivo AA was carried out as previously described for pig, bovine, canine, feline, and human eyes [17, 18, 19, 20]. Prior to imaging, eyes were wrapped in phosphate‐buffered saline (PBS) soaked gauze in a sealed container and stored at 4°C. Orientation of superior, inferior, nasal, and temporal quadrants in each eye was verified based on the direction of the attached optic nerve, the course of the long posterior ciliary arteries, and the position of the nictitating membrane prior to pinning the remaining adnexal tissue to soft modeling clay.

Briefly, a 20‐gauge Lewicky anterior chamber maintainer (ACM; Accutome, Malvern, Pennsylvania) was inserted into the anterior chamber through a 22‐gauge side‐port incision in the limbal cornea and connected to a fluid reservoir containing Lactated Ringer's Solution (LRS; Baxter Healthcare Corporation, Deerfield, IL). The location of the ACM was consistently assigned to the 10 o'clock position, which corresponded to the superiotemporal and superionasal quadrants of the right and left eyes, respectively. For both experimental groups of eyes, the height of the LRS reservoir was adjusted to a set IOP within a given range, and IOPs were validated with rebound tonometry using a TonoVet tonometer (Icare, Finland) [21]. The corneas were kept moist by frequent topical application of balanced salt solution (BSS) every 3–5 min, and AA was performed using the Spectralis HRA + OCT (Heidelberg Engineering Inc., Carlsbad, CA) with an anterior segment module lens.

**FIGURE 1:**
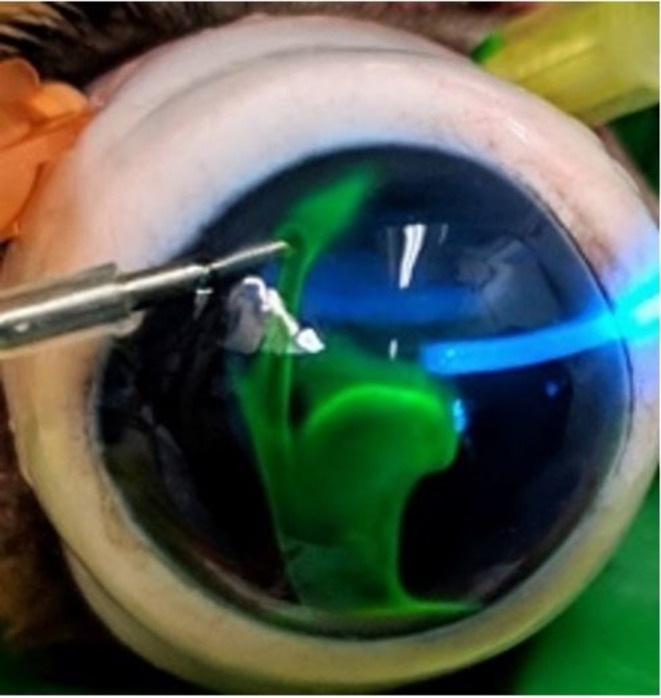
Cannulated ex vivo canine eye during infusion of Indocyanine Green tracer. Imaging was conducted by concurrent optical coherence tomography and confocal scanning laser ophthalmoscopy beginning as soon as the anterior chamber was filled with tracer.

Two experimental groups were studied: in the first group of eyes (*n* = 12), the height of the LRS reservoir was adjusted as necessary to maintain an IOP between 10 and 25 mmHg (canine physiological pressure) for 30 min. Following the 30‐min perfusion period, 2.5% fluorescein (Akorn Inc., Illinois) in BSS was infused into the anterior chamber at physiologic pressure (Group 1A). Concurrent confocal scanning laser ophthalmoscopy (cSLO) and OCT image acquisition began immediately after tracer infusion, and images were acquired in each quadrant of the sclera at approximately 2‐min intervals for 15 min. All OCT scleral line scan images were taken at 90° to the limbus. After the first round of image acquisition, IOPs for the same 12 eyes were maintained at 55 to 68 mmHg (elevated pressure) for an additional 30 min (Group 1B). Following the second 30‐min perfusion period, 0.4% Indocyanine green (ICG; Diagnostic Green LLC, Ohio) in sterile water was infused into the anterior chamber of the eye (Figure 1). ICG AA images were acquired in the same manner as with fluorescein; however, images were acquired over a 30‐min period.

In a second experimental group of eyes (Group 2; *n* = 7), the height of the LRS reservoir was adjusted to maintain an IOP between 60 and 71 mmHg for 120 min. In Group 2, AA was performed with ICG in four eyes and fluorescein in three eyes according to the availability of the fluorescent tracer. Images for Group 2 were acquired in the same manner as described for Group 1A and B above.

### 2.2 OCT Measurements

Image analysis and measurement of scleral lumen heights (SLH) were done using Fiji (Image J 1.52i; National Institutes of Health, https://imagej.nih.gov/ij/) by two observers. Measurements of SLH were used instead of area because when analyzing tubular structures in cross‐sections, the area can be highly affected by small changes in scan angle and tissue orientation during image acquisition, which in turn can yield a more oblique section through a specific vessel.

**FIGURE 2:**
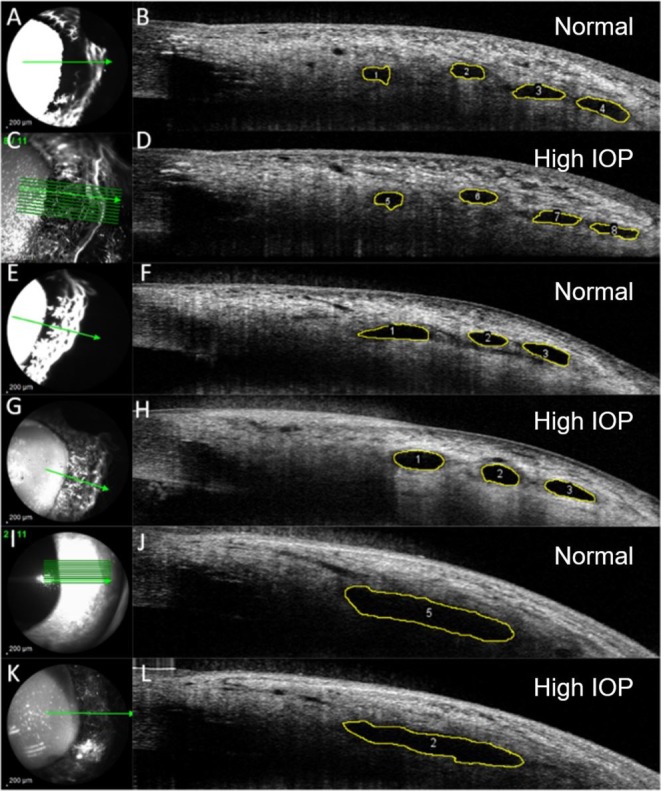
Representative aqueous angiography (AA; left) and optical coherence tomography (OCT; right) scans in normal dog eyes from experimental Group 1. Anterior segment OCT scans were performed simultaneously with AA using fluorescein (A and E) or Indocyanine Green (C, G, K) imaged by confocal scanning laser ophthalmoscopy. Image obtained with infrared light (I, shown here for reference). Heights of scleral lumens [outlined in yellow] were measured using image J in OCT scans acquired at physiologic IOP (B, F, J, “Normal”) and compared to their corresponding heights in OCT scans acquired after 30 min of elevated IOP (D, H, L, “High IOP”).

**FIGURE 3:**
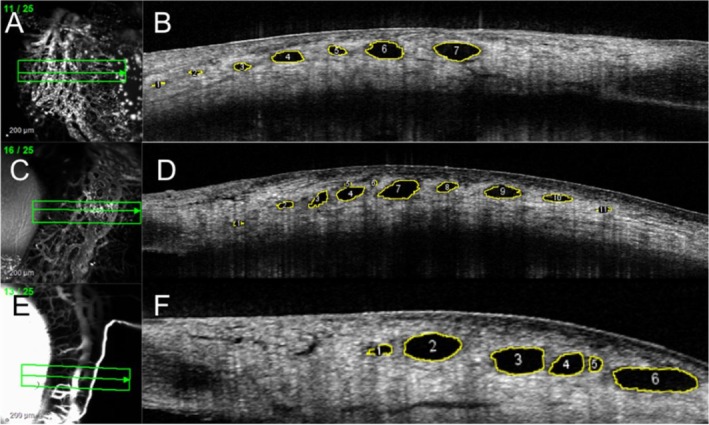
Representative aqueous angiography (AA) and Ocular Coherence Tomography (OCT) scans of healthy dog eyes from experimental Group 2. Anterior segment OCT scans were performed simultaneously with AA using Indocyanine Green (A and C), or with fluorescein (E). Scleral lumen height measured after 120 min of elevated IOP (B, D, F) was compared to values for vessels measured at physiologic IOP in experimental Group 1A eyes. (Scleral lumens outlined in yellow; Image J).

For six eyes in Group 1, at physiologic (1A) and elevated pressures (1B), SLH was quantified in the two quadrants opposite to the cannulation site (i.e., in the superotemporal and inferotemporal quadrants for left eyes and superonasal and inferonasal quadrants for right eyes). For the remaining six eyes in Group 1, at each pressure, SLH was quantified in all four quadrants. For each eye in Group 2 at elevated IOP, SLH was evaluated in all quadrants. Four non‐contiguous images were selected from each quadrant for SLH measurement depending on the feasibility of identifying the exact same lumens in cross‐section after IOP was elevated. In Group 1, a total of 1066 images were acquired; from these, 204 images were selected for SLH analysis. In these images, 249 individual vessels were measured at both IOPs (total of 498 measurements), as shown in Figure 2. The distribution of vessels analyzed per quadrant was as follows: superotemporal, *n* = 67; inferotemporal, *n* = 92; superonasal, *n* = 37; and inferonasal, *n* = 53. The total number of individual vessels analyzed per eye in Group 1 is summarized in Table 1. In Group 2, 2722 images were acquired, and 148 images were selected for SLH analysis. A total of 561 vessels were measured in these images, as shown in Figure 3. The distribution of the vessels analyzed per quadrant in eyes in Group 2 was as follows: superotemporal, *n* = 154; inferotemporal, *n* = 199; superonasal, *n* = 99; and inferonasal, *n* = 109. The total number of vessels analyzed per eye in Group 2 is summarized in Table 2.

**TABLE 1:** Group 1A eyes and corresponding number of vessels for which scleral lumen heights (SLH) were determined.

Eye	# vessels analyzed for SLH
1L	8
2R	5
2L	19
3R	41
3L	38
4R	23
4L	34
5L	8
6R	23
6L	18
7R	16
7L	16

**TABLE 2:** Group 2 eyes and corresponding number of individual vessels for which scleral lumen heights (SLH) were determined.

Eye	# vessels analyzed for SLH
8R	74
8L	32
9R	94
9L	99
10L	74
11R	97
11L	91

Additionally, in a subset of 10/12 eyes from Group 1, between 2 and 4 quadrants were subjectively characterized as being high‐flow or low‐flow regions based on angiographic signal following either fluorescein or ICG intracameral infusion, at 2 and 10 min post‐infusion, respectively. High‐flow regions were classified as having angiographic signal in ≥ 75% of the individual quadrant after the specified time frame.

### 2.3 Statistical Analysis

A reproducibility analysis was done to determine the alignment of the observers. The primary goal of this analysis was to determine if there was any bias between the two observers.

The differences in average SLH in each eye after 30 min of exposure to increased IOP were compared using a paired *t*‐test for all eyes in Group 1, allowing for the assessment of within‐subject changes after a 30‐min exposure to high IOP. Additionally, an unpaired *t*‐test was employed to compare SLH measurements at physiological IOP in Group 1 to SLH measurements after 120 min IOP elevation in Group 2. These tests were performed using GraphPad Prism version 8.4.3.

To account for other important variables and to more comprehensively assess dynamic responses of these scleral vessels, the change in SLH in Group 1B (30 min of elevated IOP) was analyzed with a linear regression model. The vessel measurements from both eyes of 5 dogs and a single eye each of another 2 dogs (for a total of 12 eyes) were included in initial analyses. Subsequent linear models used measurements from one eye per dog to account for potential between‐eye correlations that could not be corrected for in these small datasets. The independent variables were dog, SLH at physiological pressure, quadrant, and the interaction between dog and the SLH at physiological pressure. The dog and quadrant variables were sum coded in the regression model, resulting in the intercept representing the expected SLH change for very small vessels, for the average dog and quadrant, and the coefficient for the SLH model representing the expected change in SLH after exposure to elevated IOP for 30 min associated with a unit increase in vessel height at physiological pressure for the average dog and quadrant.

The change in vessel height from physiological pressure (Group 1A) after 120 min of elevated IOP (Group 2) employed different dogs and eyes for the measures acquired under the two different IOP conditions. The vessel height was analyzed using a linear regression model that utilized mean SLH values of one eye per dog. The independent variables were: an indicator for which IOP the measurement was obtained at, individual dog, and quadrant. The coefficient for the IOP indicator is the expected difference associated with a change from physiological IOP to the measurement taken after 120 min exposure to high IOP. Statistical analysis was performed using R version 4.1.1.

## 3 Results

Segmental AA outflow signal correlated with lumens seen on OCT scleral line scans in healthy canine eyes at physiologic IOP (Figure 2). These lumens were also observed in both groups at elevated IOP but appeared collapsed in profile (Figure 3). No bias between the two observers measuring vessel SLHs was identified. The test for bias between the two readers had a *p*‐value of 0.06 (bias: −2.1 μm, SD: 20.30 μm).

**FIGURE 4:**
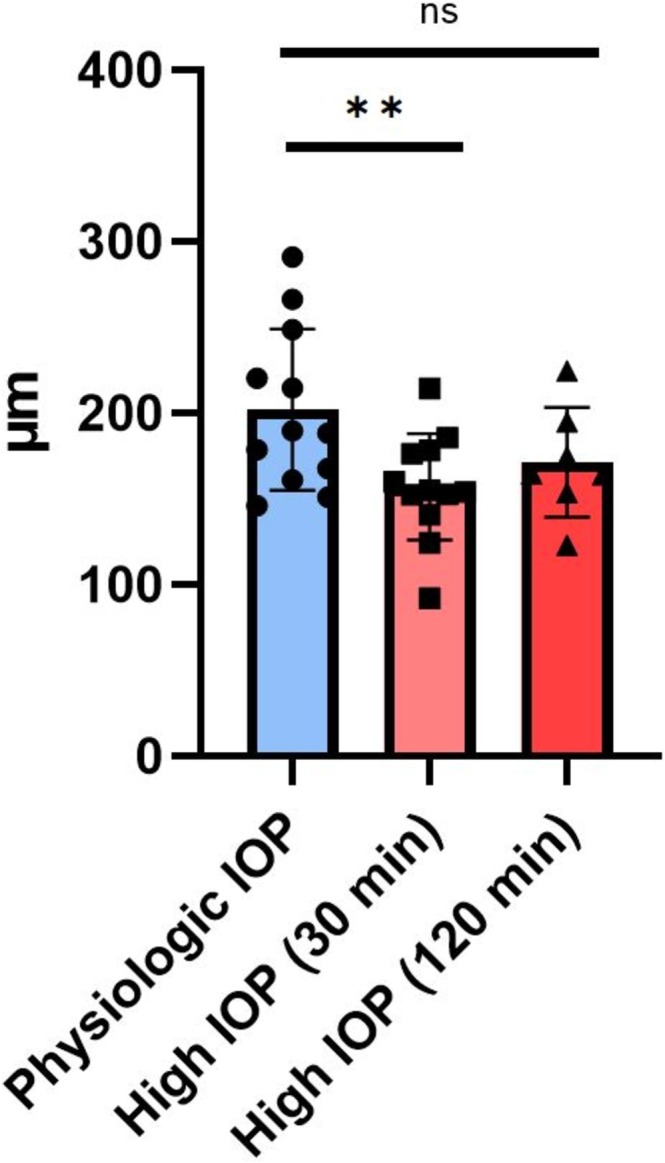
Mean scleral lumen height (SLH; μm) measured in OCT scans at physiologic IOP compared to measurements acquired after 30 and 120 min at high IOP. Error bars depict standard deviation. Each bar chart represents the mean, with the error bars showing the SEM, and each data point represents a grand average of scleral lumen height (SLH) measurements for an individual eye. **p < 0.01 (paired *t*‐test); ns = not significant (*p* > 0.05, unpaired *t*‐test).

The results of the within‐group paired *t*‐test revealed a statistically significant decrease in average SLH in Group 1B after 30 min of exposure to increased IOP (mean [± standard deviation] SLH at physiological IOP [Group 1A] = 202.102 ± 46.96 μm; mean SLH after 30 min of increased IOP [Group 1B] = 157.315 ± 31 μm. *p* = 0.0031). The mean SLH in Group 2 (171.5 ± 32.08 μm), which underwent 120 min of exposure to high IOP, was similar to that of Group 1B after 30 min of exposure to high IOP, but the unpaired *t*‐test comparing SLH between Groups 1A and 2 did not reach statistical significance (*p* = 0.1). A summary plot of mean SLH of vessel lumen height data in each eye, at physiological IOP and after 30 and 120 min of elevated IOP, is presented in Figure 4.

**FIGURE 5:**
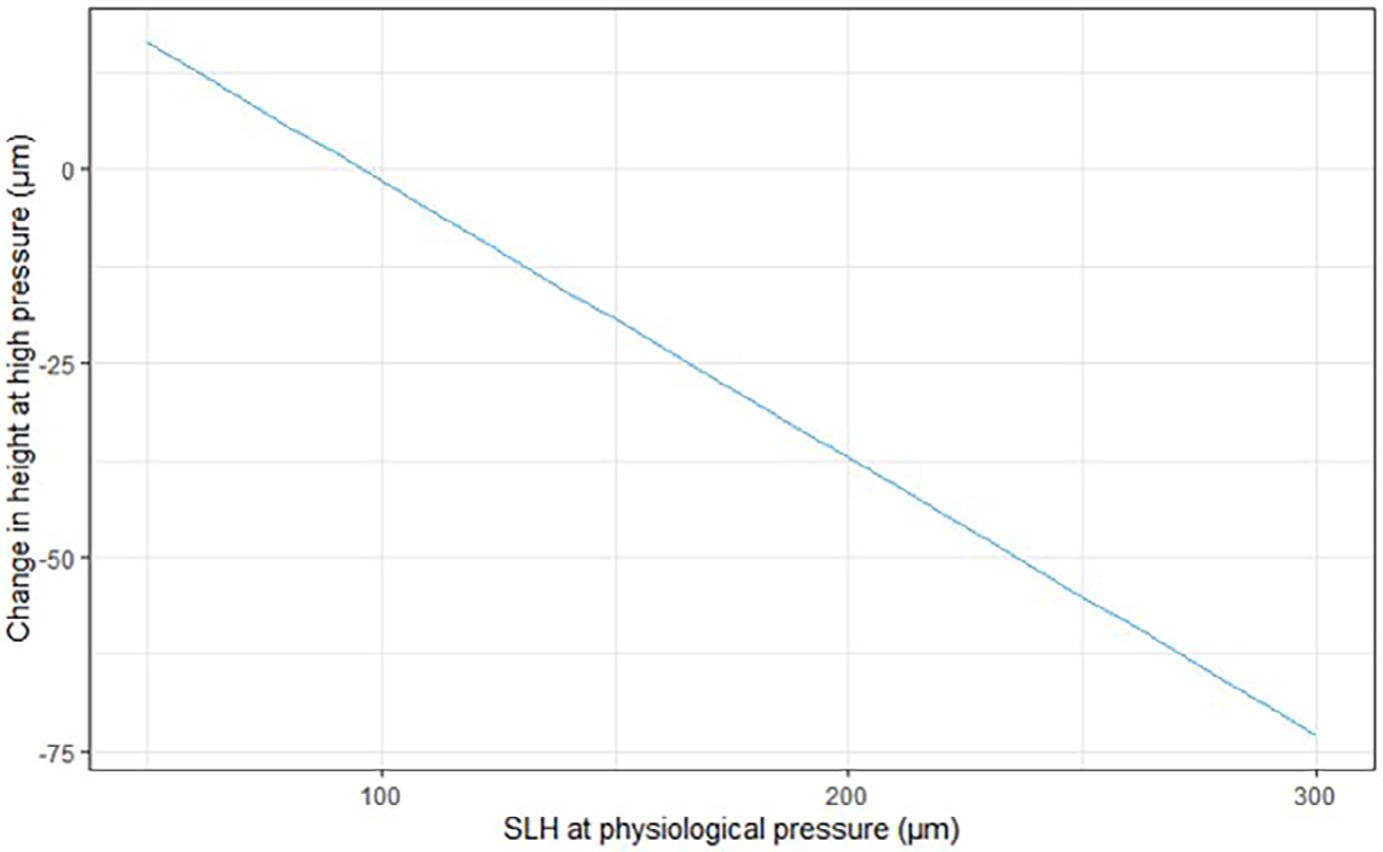
Plot displaying estimated expected change in scleral lumen height (SLH) between measurements at physiological pressure and measurements at high pressure (30 min) for the average dog eye in experimental Group 1 fitted to a linear model.

In the linear model for experimental Group 1, larger scleral lumens at physiologic IOP showed a greater reduction in SLH following exposure to 30 min of elevated IOP (intercept: 34.785, coefficient: 0.643; both, *p* < 0.05). That means that the value of the difference between SLH after 30 min of elevated IOP and initial SLH at physiological IOP was increasingly negative for vessels that had a larger SLH at physiological pressure. In other words, larger vessels collapse more than small vessels after exposure to high IOP for 30 min, while smaller vessels may even expand in response to 30 min of IOP elevation in normal eyes. This phenomenon can be seen in the linear regression model presented in Figure 5 (for data see Supplemental file 1). From this figure, one can observe that there is little or no change in SLH after 30 min of elevated IOP in those vessels that have a SLH < ~100 μm at low physiologic IOP. However, as SLH increased above about 100 μm at physiologic IOP, the SLH of these larger vessels was reduced after a 30‐min exposure to high IOP. No individual dog or eye had a significant departure from this expected change, and there were no individual quadrants with departure from the mean values calculated for vessels across all sectors.

The expected change in SLH comparing measurements from Group 1 eyes exposed to physiologic IOP and measurements from Group 2 eyes subjected to a 120‐min period of high IOP was similar to that observed after a 30‐min exposure to high IOP, but the decrease in SLH was more pronounced (intercept: 222.952, coefficient: −115.809; both *p* < 0.0001). The intercepts and coefficients for SLH in all experimental groups are summarized in Table 3.

**TABLE 3:** Intercept, coefficient, and *p*‐values of the linear models for each group.

	30‐min IOP increase	120‐min IOP increase
Intercept	34.782 (±27.88)	222.952 (±47.56)
*p*‐value	0.0149	< 0.0001
Coefficient	0.643 (±0.13)	−115.809 (±49.3)
*p*‐value	< 0.0001	< 0.0001

*Note:* Standard deviation (SD) is indicated in parentheses. The intercept represents the expected SLH after high IOP exposure for a vessel with a physiologic SLH of 1 μm for the average dog eye. The coefficient represents the expected change in SLH for a unit increase in IOP.

Subjective analysis of aqueous angiographic signal intensity (Table 4) suggests that elevated IOP results in a decrease in circumferential, high‐flow segmental aqueous humor outflow as indicated by a decrease from a total of 14 high‐flow regions identified at physiologic IOP to just 8 high‐flow regions in the same eyes at elevated IOP. No low‐flow quadrants were observed to convert to high‐flow after elevation of IOP. Additionally, superior quadrants were identified as low flow more frequently at physiologic (14/16) and elevated IOP (15/16) compared to corresponding inferior quadrants at physiologic (4/16) and elevated IOP (8/16), respectively.

**TABLE 4:** Subjective analysis of segmental aqueous humor outflow signal, in a subset of 10 eyes before (1A) and after 30 min of IOP elevation (1B).

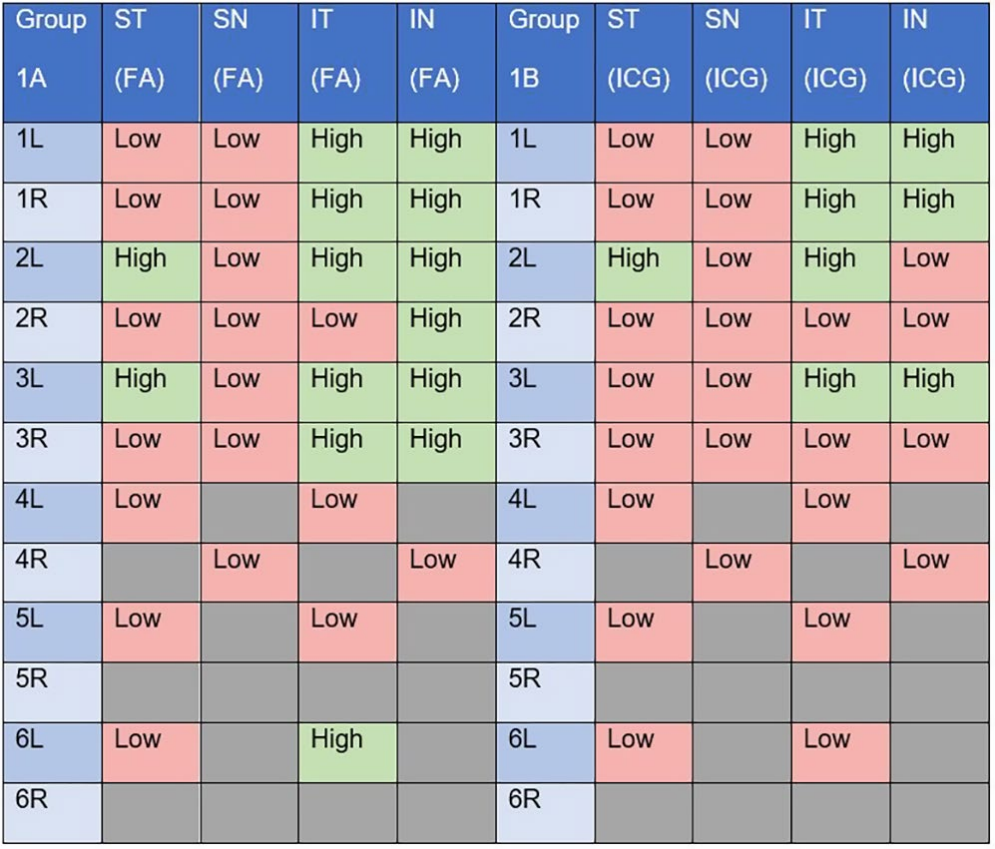

*Note:* Assessment was based on intensity of signal from fluorescein (FA; Group 1A) and Indocyanine Green (ICG) (Group 1B) in confocal laser scanning ophthalmoscope images. High flow (green boxes) was defined as a quadrant with aqueous angiography signal in ≥ 75% of the area within 2 min for FA and 10 min for ICG, versus low flow (red boxes). ST (superiotemporal), SN (superionasal), IT (inferiotemporal), and IN (inferionasal). Grey boxes indicate quadrants for which an insufficient sample and/or quality of images were available for assessment.

## 4 Discussion

There are relatively few published studies in human and veterinary medicine evaluating the distal outflow pathway in glaucomatous eyes [16, 17, 20, 22]. A previous study identified differences in scleral vascular channels and absence of AA signal in canine eyes with PACG [17]. Lack of angiographic signal in canine PACG likely reflects complete obstruction of the proximal outflow structures, including the trabecular meshwork, by extensive peripheral anterior synechiae and angle closure [17, 23, 24]. However, whether collapse of scleral vascular channels represents a cause or effect of elevated IOP was not established in that study. In the current study, normal canine eyes were used to investigate the potential causal relationship between increased IOP and structural changes previously observed in the post‐trabecular aqueous outflow tract of dogs enucleated for PACG. Our findings indicate that even in normal eyes, pathologic increases in IOP can affect the post‐trabecular outflow pathway, inducing morphologic changes in scleral lumens and an overall reduction in SLH. However, in these normal canine eyes ex vivo, there would still be aqueous humor outflow through the TM and AAP upstream of the scleral lumens. The apparent mitigation of acute IOP‐mediated reduction in SLH in some of the smaller vessels by longer IOP elevation/LRS infusion could potentially be explained by the “washout” phenomenon, which has been reported in dog eyes [25, 26]. In contrast, in PACG eyes in the previous study, there was probably no meaningful proximal outflow facility upstream of the distal vessels imaged, thus preventing any “washout”‐related increase in outflow to the distal vessels. While we demonstrated that elevated IOP significantly decreased the SLH of distal aqueous outflow vessels in normal canine eyes, we were unable to establish a direct association between the magnitude of this change in the cross‐sectional height of vessels and the duration of exposure to elevated IOP. The discrepancy between the unpaired *t*‐test and linear model results may be due to the linear model's ability to account for additional variables, providing a more nuanced understanding of vessel dynamics after extended high IOP exposure. The limited power of the unpaired *t*‐test due to the relatively small sample size and the lack of paired measurements at physiological pressure may have also contributed to the observed discrepancy. A paired design for Group 2 could have provided more insight and increased power to detect differences, even with the same sample size. However, this proved impractical in the current study due to time constraints imposed on us by the opportunistic nature of our acquisition of eyes at the conclusion of other investigators' studies. We acknowledge that our study design emphasized structural changes but did not rigorously control IOP, which would have required more precisely calibrated, constant pressure perfusion experiments. We did not evaluate important functional metrics of aqueous humor dynamics, including outflow facility and relative contribution of the washout phenomenon, which likely enhanced conventional outflow through the trabecular meshwork in these normal canine eyes in the current study. Thus, an important limitation and potential confounder in our study is that the washout phenomenon—whereby outflow resistance decreases with prolonged perfusion in canine eyes—could not be directly controlled for. However, the washout phenomenon typically increases proximal outflow and would be expected to increase filling of distal structures, which contrasts with our observed collapse of distal outflow structures under elevated IOP. Since our study focused on isolating the effects of both acute and more sustained IOP elevation intended to mimic IOPs in PACG, our inability to control for the confounding role of washout in canine aqueous humor outflow does not completely negate our findings. Future studies would be strengthened by more rigorous attempts to account for dynamic resistance changes in the more proximal structures of the conventional outflow pathway.

Previous studies on the TM and proximal aqueous humor outflow pathway have demonstrated pressure‐dependent morphological changes in normal non‐human primate and bovine eyes. These changes include a decrease in TM displacement, an increase in stiffness, and a reduction of the size of Schlemm's canal and AAP, as well as changes in vacuolation of the endothelium [27, 28, 29, 30]. In the current study, AA and OCT imaging emphasized more distal components of this conventional aqueous outflow pathway, through which 85%–90% of the aqueous humor exits the anterior chamber in normal dogs [31]. Findings in the current study are consistent with studies evaluating the effects of IOP elevation in human eyes, which demonstrate a reduction in the cross‐sectional area of Schlemm's canal [32]. Moreover, compression of Schlemm's canal in response to IOP elevation has been reported in mathematical modeling studies [33], while another study found that increasing the diameter of the luminal cross‐sectional area of Schlemm's canal of human eyes resulted in a decrease in IOP [34]. Unfortunately, limitations on the resolution of the imaging techniques utilized in the current study precluded assessment of the AAP, the canine equivalent to Schlemm's canal.

Several other limitations of our study are acknowledged. Fluorescein and ICG differ in key physicochemical properties that influence their performance in aqueous angiography. Fluorescein (376 g/mol) has a lower molecular weight and protein binding (70%–90%), leading to faster visualization but greater leakage from aqueous humor outflow (AHO) pathways. ICG (775 g/mol) exhibits near‐complete protein binding (98%), better intraluminal retention, and reduced extravasation, resulting in sharper anatomical delineation despite slower signal development. Their spectral properties also differ: fluorescein emits in visible light (525 nm), while ICG uses near‐infrared wavelengths (835 nm), enabling deeper tissue penetration [35, 36]. Fluorescein was used prior to ICG in eyes undergoing two rounds of AA; however, it may be beneficial to utilize ICG prior to fluorescein due to potential interference with the signal [18]. However, when ICG was administered following fluorescein, we did not observe any fluorescein signal in the AA field prior to visualization of ICG, and ICG was administered following extended perfusion with LRS for either 30 or 120 min. Furthermore, the intensity of the AA signal was only evaluated subjectively as a secondary outcome in this study, and we characterized crude patterns and timing of signal appearance within scleral lumens only as a surrogate for the presence of *any* aqueous humor outflow into these lumens, rather than as a quantitative measure of aqueous outflow facility. Objective, quantitative analysis of signal intensity or direct quantitation of aqueous humor flow rates (outflow facility) was not undertaken in the present study. Thus, the order in which tracers were administered did not appear to impact our results.

Though angiographic signal was consistently associated with more high‐flow regions in the inferior quadrants, it is conceivable that this finding may be gravity dependent rather than representative of an inherent biological property of the tissues in this region. There are substantial limitations of our crude, quadrant‐based assessment, which was chosen for simplicity of this secondary analysis. Despite evidence from other studies that outflow regions can be smaller than quadrant‐sized segments, the regions of high and low outflow were relatively large in our study and reasonably well captured by this simplistic approach, as representative images in Figure 2E,K, respectively, illustrate. Previous studies using particulate tracers have identified micro‐scale high/low‐flow zones, but to achieve the overarching goal of our study, we essentially set out to use aqueous angiography to register specific vessels for OCT imaging. We acknowledge that re‐analysis at finer scales could yield greater insight, but quadrant‐level data provided a pragmatic starting point given the inherent limitations posed by relatively small sample sizes in our study. Additional experiments with globes oriented differently during testing and more rigorous and granular characterization of smaller, defined regions as high flow or low flow could help clarify the relationship between distal aqueous outflow signal and the effects of gravity in this ex vivo model. Importantly, this ex vivo study does not fully recapitulate in vivo conditions, due to a lack of distal venous pressure as well as *postmortem* impairment of energy dependent processes that contribute to aqueous humor outflow regulation in vivo.

Additionally, statistical analyses in experimental Group 2 eyes after 120 min of high IOP did not include measurements of the same vessels before and after increasing IOP for a direct comparison. As a result, our analyses assumed that the SLH of the vessels in Group 2 at normal IOP was similarly distributed to that of the vessels measured in the eyes of Group 1A. There were some particular quadrants and particular dog eyes that showed both subjective and objectively clear and significant differences in their degree of SLH reduction and collapse compared to corresponding quadrants of average “reference” dog eye (with the “reference” being considered the average trend within the group of tested eyes) used to create the statistical model. Differences such as these were expected, however, and reflect the potential for significant variations between individual dogs and between different areas of the same dog eye.

In conclusion, acute IOP elevation resulted in a significant reduction in SLH in healthy canine eyes. However, acute IOP elevation alone is likely not solely responsible for the near complete lack of perfused scleral lumens and lack of AA signal reported previously in canine eyes with PACG. Future studies to better understand the nature and timing of this pressure‐dependent change in the post‐trabecular outflow pathway in glaucomatous eyes, including its potential reversibility, will be crucial in developing novel diagnostic tools and treatments for canine glaucoma.

## Ethics Statement

This study involved cadaveric animal tissues obtained from animals that were euthanized for other reasons unrelated to the current study, with approval by the Institutional Animal Care and Use Committee as appropriate. This study complies with the Guidelines for Ethical Research in Veterinary Ophthalmology (GERVO) and is exempt from approval by an ethics committee.

## Conflicts of Interest

The authors declare no conflicts of interest.

## Supporting information


**Appendix S1:** vop70080‐sup‐0001‐AppendixS1.docx.

## Data Availability

The data that support the findings of this study are available from the corresponding author upon reasonable request.
