# Supplementary material for: Imaging Canine Post‐Trabecular Aqueous Outflow Pathways: Effect of Acute Intraocular Pressure Elevation in Normal Eyes
Source: Vet Ophthalmol. 2025 Sep 22;29(2):e70080. doi: 10.1111/vop.70080 (PMC12744534; doi:10.1111/vop.70080)
Supplement: Supplementary file 1 — Appendix S1: vop70080‐sup‐0001‐AppendixS1.docx. [file VOP-29-0-s001.docx]

Supplemental File:

| Low pressure Hig  50.00 | h pressure  66.30 | Change High pressure log Change log  16.30 55.07 5.07 | | |
| --- | --- | --- | --- | --- |
| 60.00 | 72.74 | 12.74 | 63.56 | 3.56 |
| 70.00 | 79.17 | 9.17 | 71.73 | 1.73 |
| 80.00 | 85.60 | 5.60 | 79.64 | -0.36 |
| 90.00 | 92.04 | 2.04 | 87.31 | -2.69 |
| 100.00 | 98.47 | -1.53 | 94.80 | -5.20 |
| 110.00 | 104.90 | -5.10 | 102.11 | -7.89 |
| 120.00 | 111.33 | -8.67 | 109.27 | -10.73 |
| 130.00 | 117.77 | -12.23 | 116.29 | -13.71 |
| 140.00 | 124.20 | -15.80 | 123.20 | -16.80 |
| 150.00 | 130.63 | -19.37 | 129.99 | -20.01 |
| 160.00 | 137.06 | -22.94 | 136.67 | -23.33 |
| 170.00 | 143.50 | -26.50 | 143.26 | -26.74 |
| 180.00 | 149.93 | -30.07 | 149.77 | -30.23 |
| 190.00 | 156.36 | -33.64 | 156.19 | -33.81 |
| 200.00 | 162.79 | -37.21 | 162.53 | -37.47 |
| 210.00 | 169.23 | -40.77 | 168.80 | -41.20 |
| 220.00 | 175.66 | -44.34 | 175.01 | -44.99 |
| 230.00 | 182.09 | -47.91 | 181.14 | -48.86 |
| 240.00 | 188.52 | -51.48 | 187.22 | -52.78 |
| 250.00 | 194.96 | -55.04 | 193.24 | -56.76 |
| 260.00 | 201.39 | -58.61 | 199.21 | -60.79 |
| 270.00 | 207.82 | -62.18 | 205.12 | -64.88 |
| 280.00 | 214.25 | -65.75 | 210.99 | -69.01 |
| 290.00 | 220.69 | -69.31 | 216.80 | -73.20 |
| 300.00 | 227.12 | -72.88 | 222.57 | -77.43 |

SUPPLEMENTAL TABLE 1: displays the estimated relationship between scleral vessel lumen height at low, physiologic intraocular pressure and at high intraocular pressure (after 30minutes) for the typical dog in Experimental Group 1. The columns that end in log are from the log transformed model.

13
